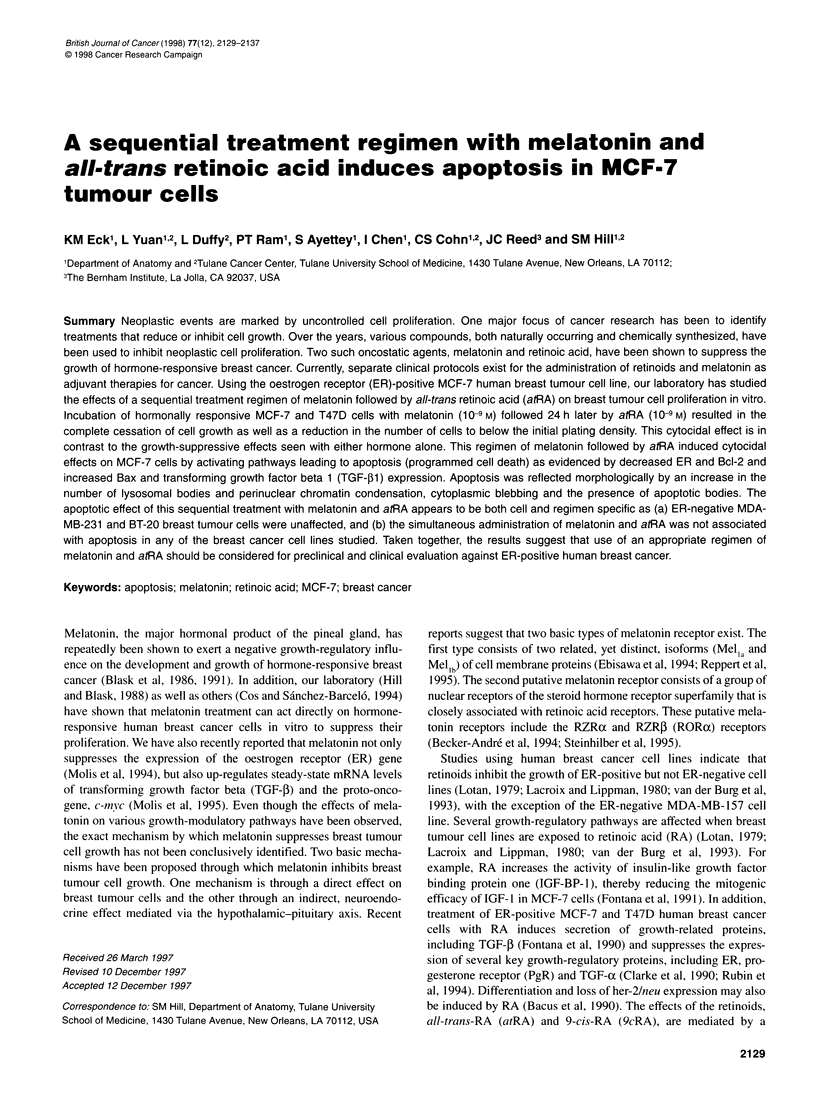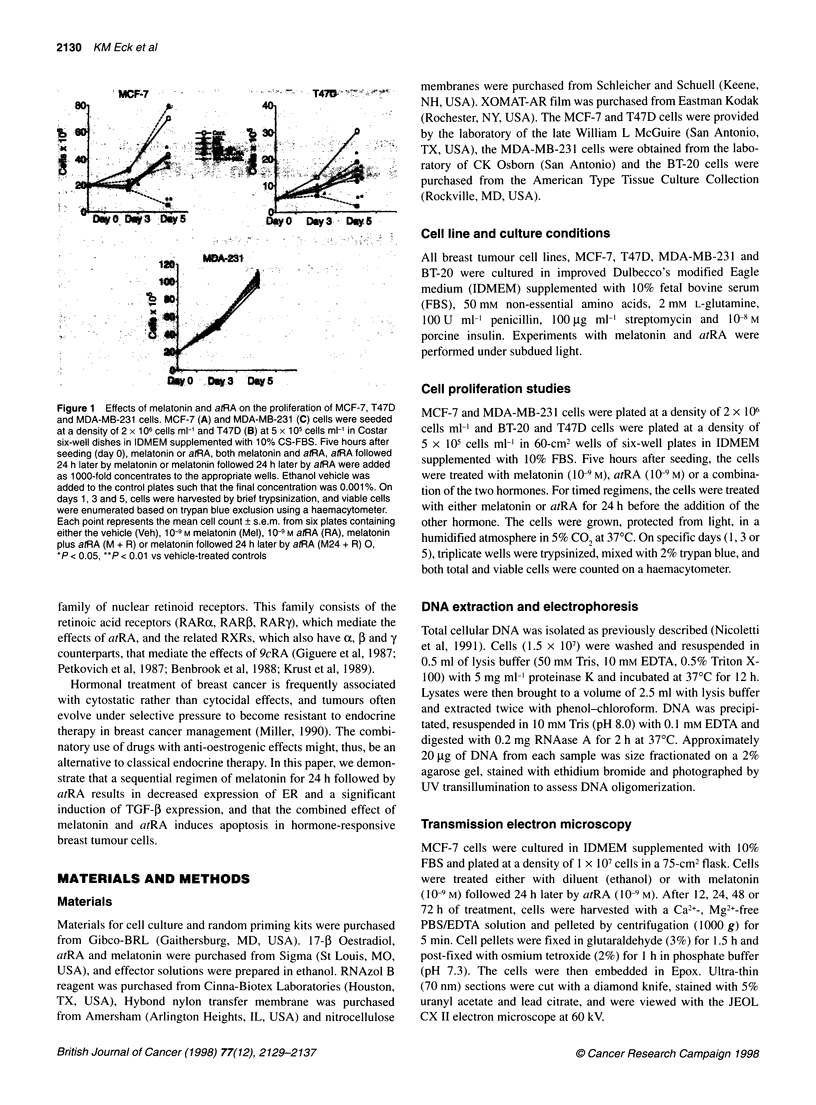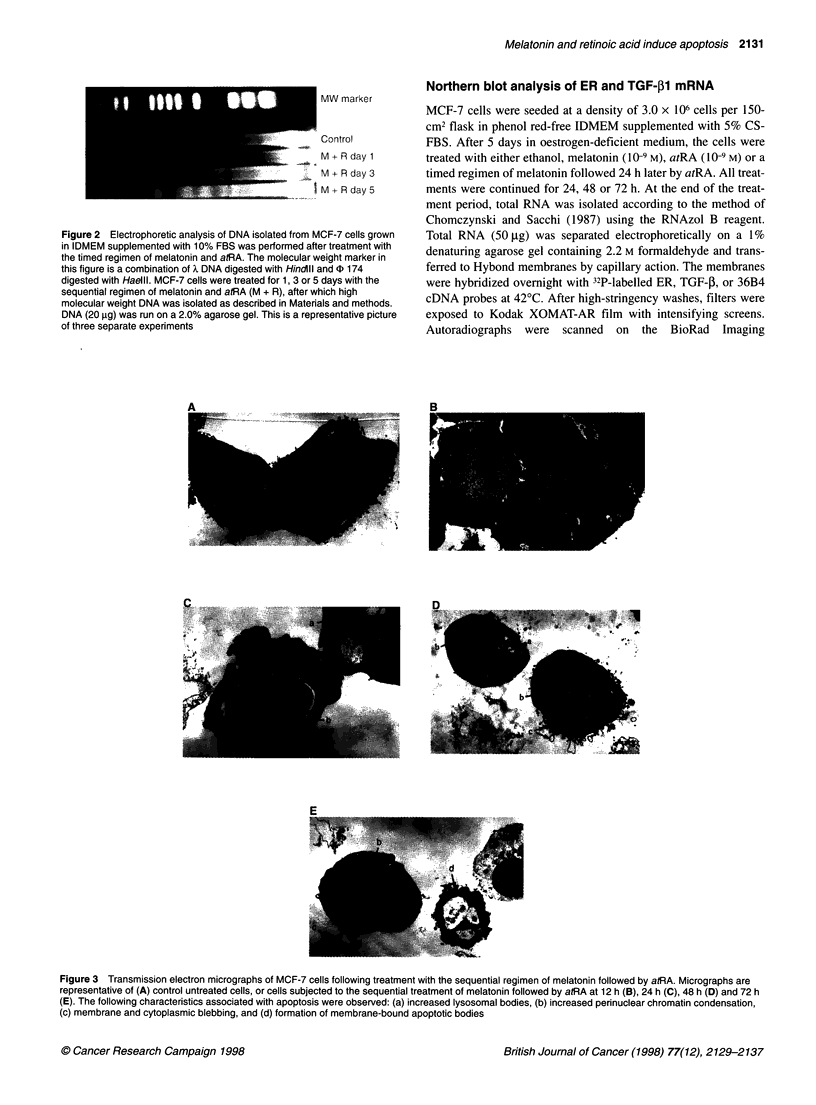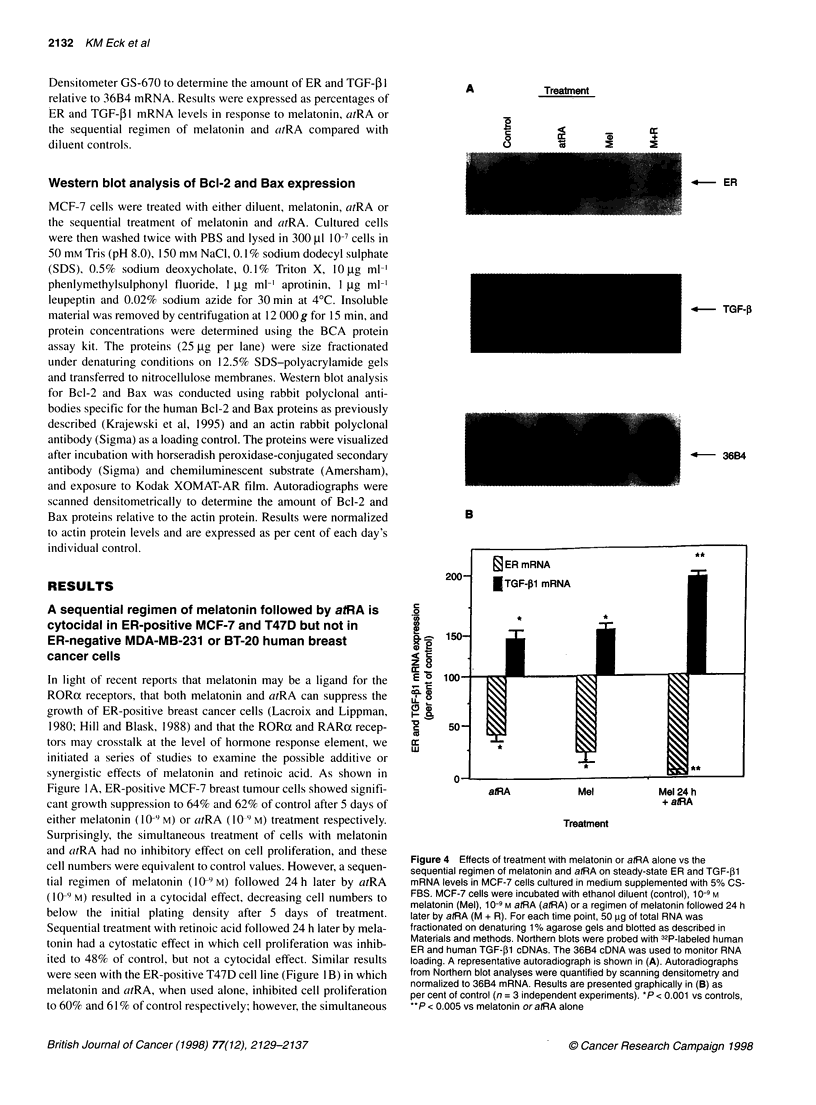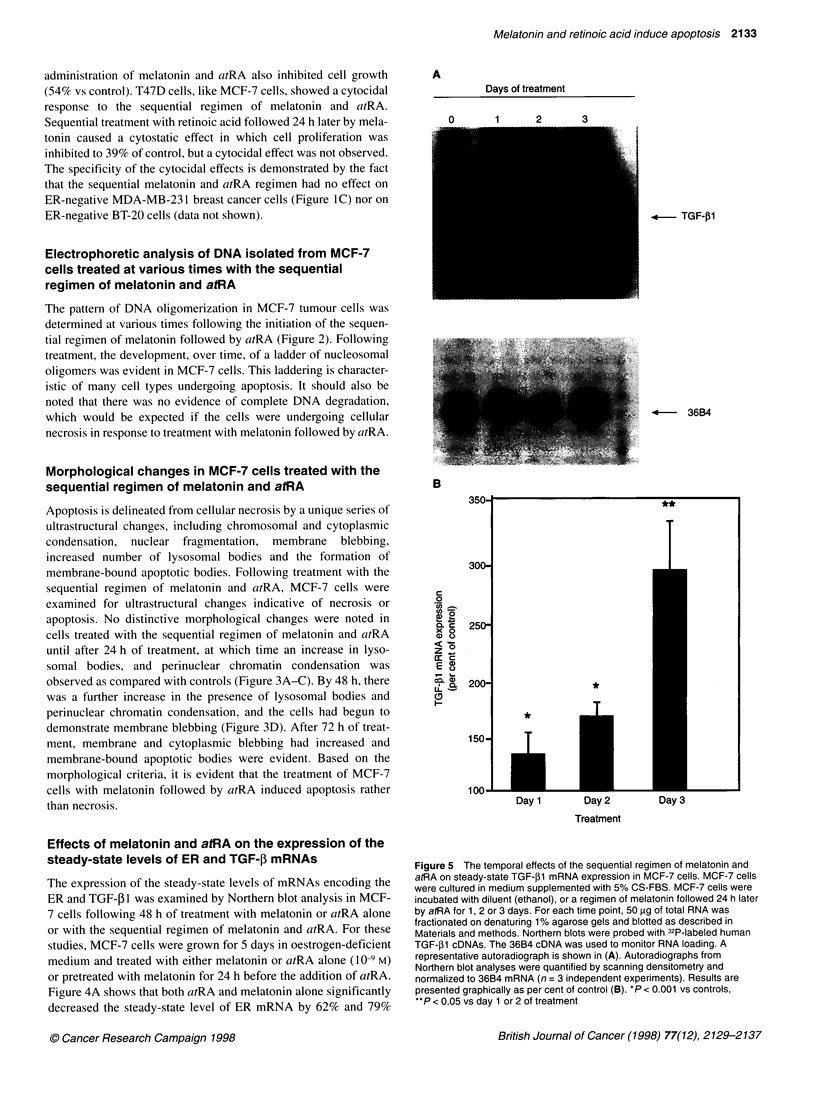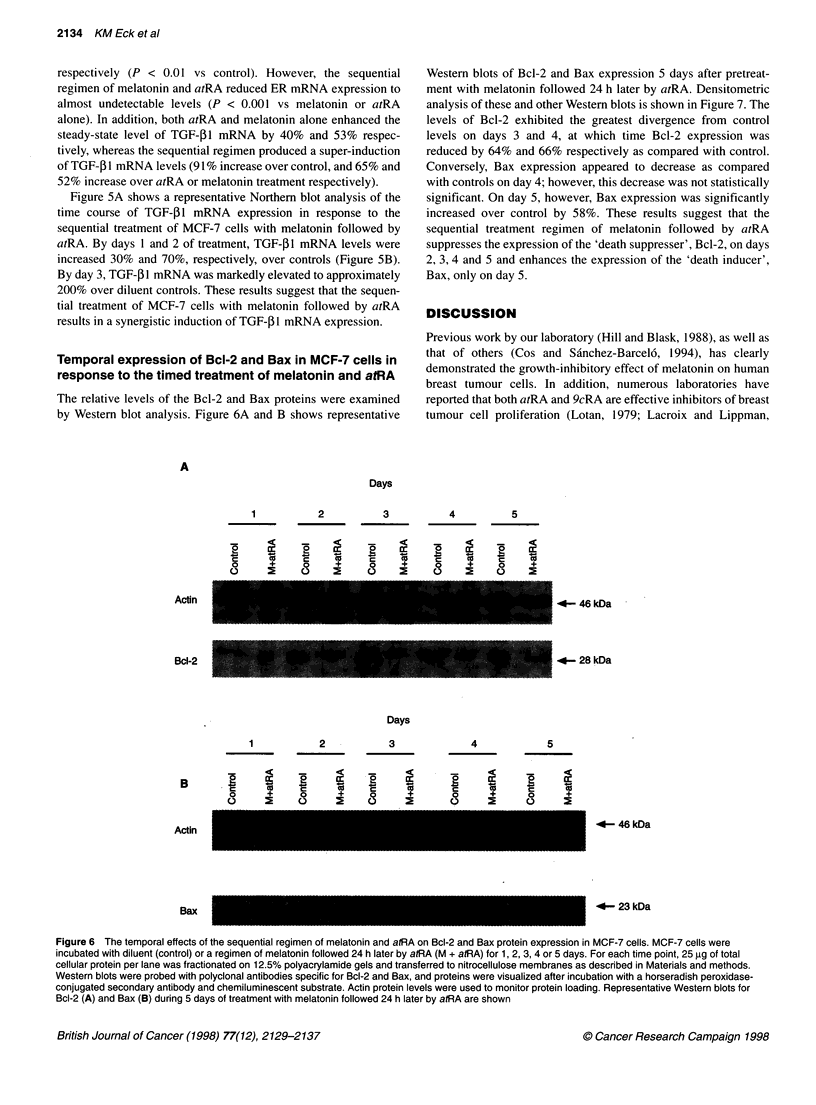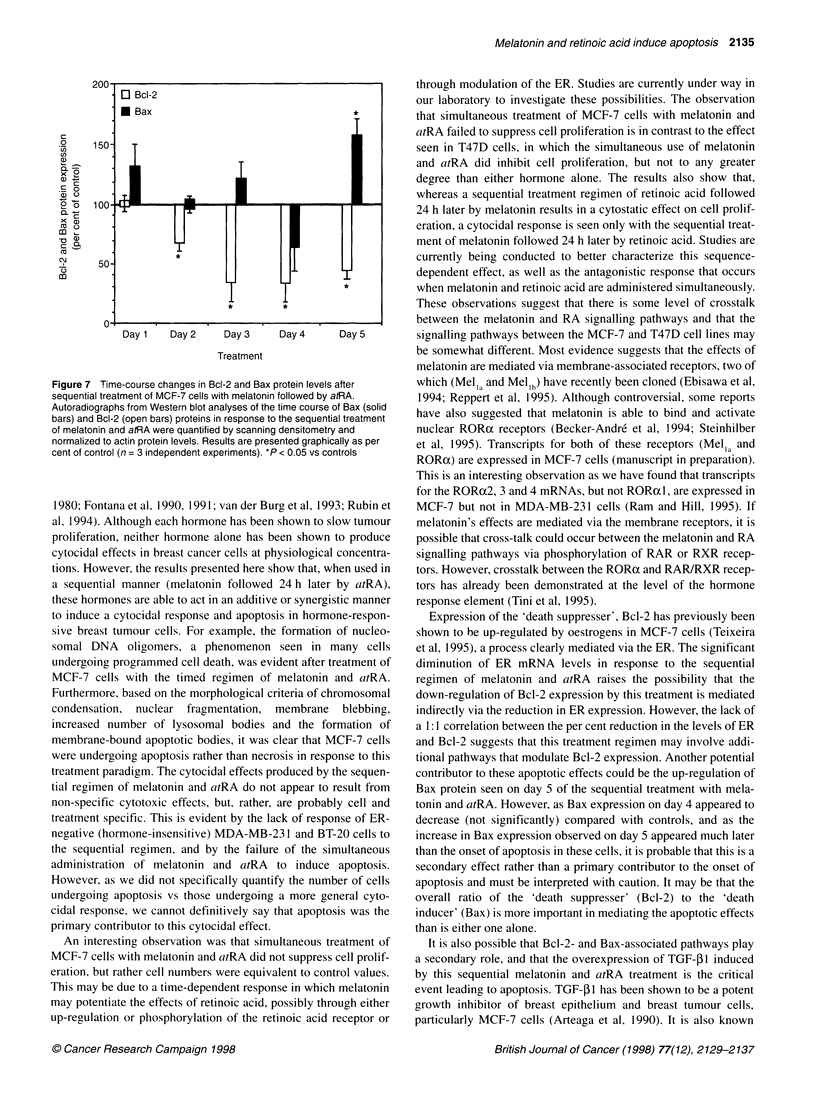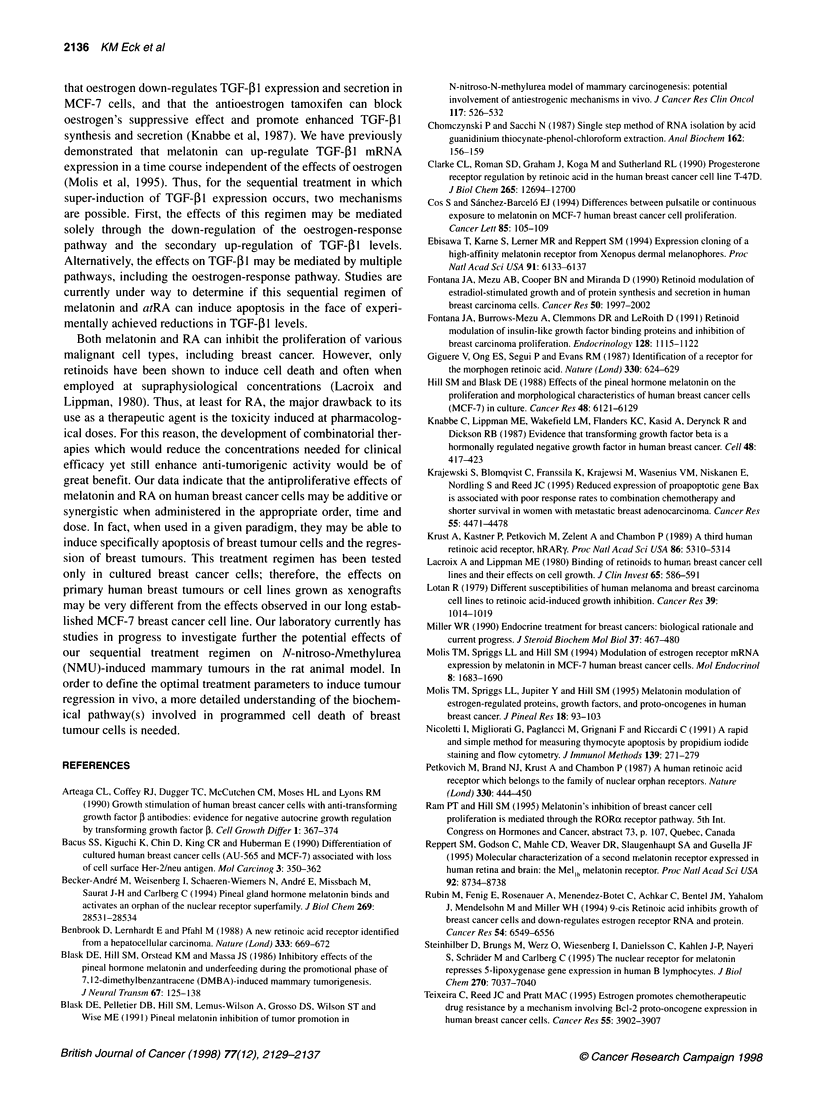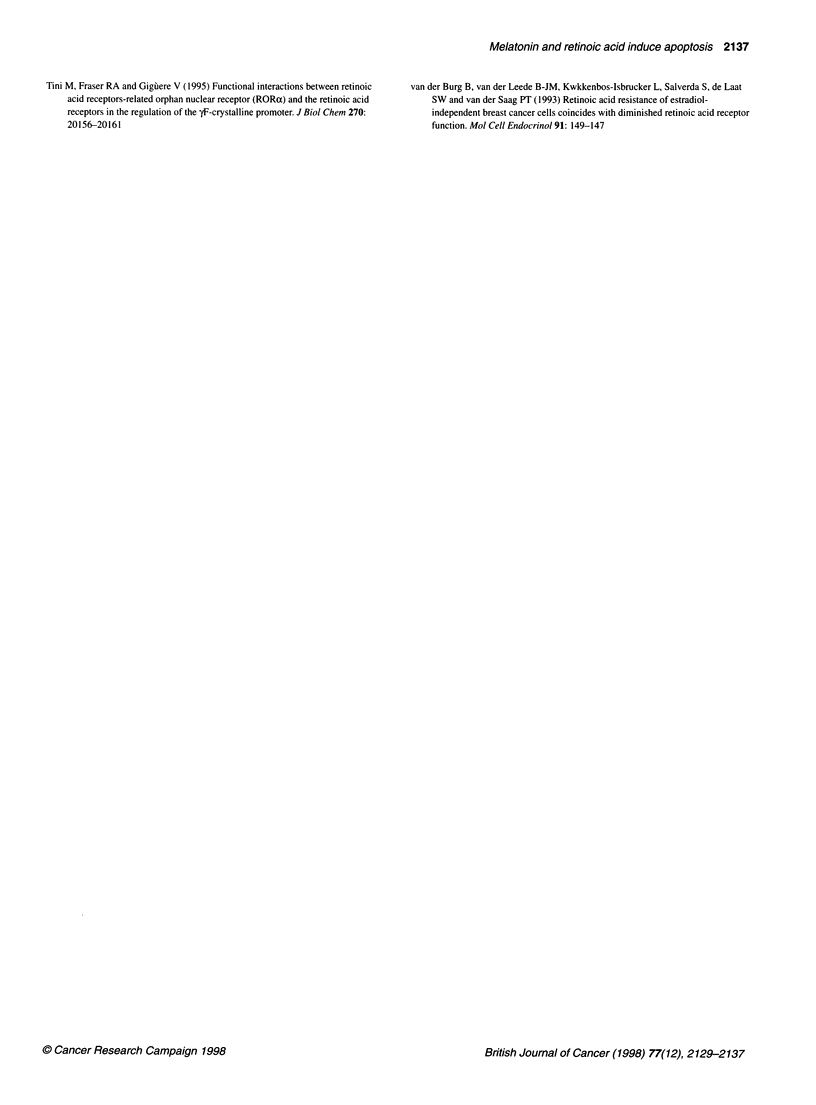# A sequential treatment regimen with melatonin and all-trans retinoic acid induces apoptosis in MCF-7 tumour cells.

**DOI:** 10.1038/bjc.1998.357

**Published:** 1998-06

**Authors:** K. M. Eck, L. Yuan, L. Duffy, P. T. Ram, S. Ayettey, I. Chen, C. S. Cohn, J. C. Reed, S. M. Hill

**Affiliations:** Department of Anatomy, Tulane University School of Medicine, New Orleans, LA 70112, USA.

## Abstract

**Images:**


British Journal of Cancer (1998) 77(12), 2129–2137
© 1998 Cancer Research Campaign

### A sequential treatment regimen with melatonin and *all-trans* retinoic acid induces apoptosis in MCF-7 tumour cells

**KM Eck[1], L Yuan[1,2], L Duffy[2], PT Ram[1], S Ayettey[1], I Chen[1], CS Cohn[1,2], JC Reed[3] and SM Hill[1,2]**

[1]Department of Anatomy and [2]Tulane Cancer Center, Tulane University School of Medicine, 1430 Tulane Avenue, New Orleans, LA 70112;
[3]The Bernham Institute, La Jolla, CA 92037, USA

**Summary** Neoplastic events are marked by uncontrolled cell proliferation. One major focus of cancer research has been to identify treatments that reduce or inhibit cell growth. Over the years, various compounds, both naturally occurring and chemically synthesized, have been used to inhibit neoplastic cell proliferation. Two such oncostatic agents, melatonin and retinoic acid, have been shown to suppress the growth of hormone-responsive breast cancer. Currently, separate clinical protocols exist for the administration of retinoids and melatonin as adjuvant therapies for cancer. Using the oestrogen receptor (ER)-positive MCF-7 human breast tumour cell line, our laboratory has studied the effects of a sequential treatment regimen of melatonin followed by *all-trans* retinoic acid (*at*RA) on breast tumour cell proliferation in vitro. Incubation of hormonally responsive MCF-7 and T47D cells with melatonin ($10^{-9}$ M) followed 24 h later by *at*RA ($10^{-9}$ M) resulted in the complete cessation of cell growth as well as a reduction in the number of cells to below the initial plating density. This cytocidal effect is in contrast to the growth-suppressive effects seen with either hormone alone. This regimen of melatonin followed by *at*RA induced cytocidal effects on MCF-7 cells by activating pathways leading to apoptosis (programmed cell death) as evidenced by decreased ER and Bcl-2 and increased Bax and transforming growth factor beta 1 (TGF-β1) expression. Apoptosis was reflected morphologically by an increase in the number of lysosomal bodies and perinuclear chromatin condensation, cytoplasmic blebbing and the presence of apoptotic bodies. The apoptotic effect of this sequential treatment with melatonin and *at*RA appears to be both cell and regimen specific as (a) ER-negative MDA-MB-231 and BT-20 breast tumour cells were unaffected, and (b) the simultaneous administration of melatonin and *at*RA was not associated with apoptosis in any of the breast cancer cell lines studied. Taken together, the results suggest that use of an appropriate regimen of melatonin and *at*RA should be considered for preclinical and clinical evaluation against ER-positive human breast cancer.

**Keywords:** apoptosis; melatonin; retinoic acid; MCF-7; breast cancer

Melatonin, the major hormonal product of the pineal gland, has repeatedly been shown to exert a negative growth-regulatory influence on the development and growth of hormone-responsive breast cancer (Blask et al, 1986, 1991). In addition, our laboratory (Hill and Blask, 1988) as well as others (Cos and Sánchez-Barceló, 1994) have shown that melatonin treatment can act directly on hormone-responsive human breast cancer cells in vitro to suppress their proliferation. We have also recently reported that melatonin not only suppresses the expression of the oestrogen receptor (ER) gene (Molis et al, 1994), but also up-regulates steady-state mRNA levels of transforming growth factor beta (TGF-β) and the proto-oncogene, *c-myc* (Molis et al, 1995). Even though the effects of melatonin on various growth-modulatory pathways have been observed, the exact mechanism by which melatonin suppresses breast tumour cell growth has not been conclusively identified. Two basic mechanisms have been proposed through which melatonin inhibits breast tumour cell growth. One mechanism is through a direct effect on breast tumour cells and the other through an indirect, neuroendocrine effect mediated via the hypothalamic–pituitary axis. Recent reports suggest that two basic types of melatonin receptor exist. The first type consists of two related, yet distinct, isoforms ($Mel_{1a}$ and $Mel_{1b}$) of cell membrane proteins (Ebisawa et al, 1994; Reppert et al, 1995). The second putative melatonin receptor consists of a group of nuclear receptors of the steroid hormone receptor superfamily that is closely associated with retinoic acid receptors. These putative melatonin receptors include the RZRα and RZRβ (RORα) receptors (Becker-André et al, 1994; Steinhilber et al, 1995).

Studies using human breast cancer cell lines indicate that retinoids inhibit the growth of ER-positive but not ER-negative cell lines (Lotan, 1979; Lacroix and Lippman, 1980; van der Burg et al, 1993), with the exception of the ER-negative MDA-MB-157 cell line. Several growth-regulatory pathways are affected when breast tumour cell lines are exposed to retinoic acid (RA) (Lotan, 1979; Lacroix and Lippman, 1980; van der Burg et al, 1993). For example, RA increases the activity of insulin-like growth factor binding protein one (IGF-BP-1), thereby reducing the mitogenic efficacy of IGF-1 in MCF-7 cells (Fontana et al, 1991). In addition, treatment of ER-positive MCF-7 and T47D human breast cancer cells with RA induces secretion of growth-related proteins, including TGF-β (Fontana et al, 1990) and suppresses the expression of several key growth-regulatory proteins, including ER, progesterone receptor (PgR) and TGF-α (Clarke et al, 1990; Rubin et al, 1994). Differentiation and loss of her-2/*neu* expression may also be induced by RA (Bacus et al, 1990). The effects of the retinoids, *all-trans*-RA (*at*RA) and 9-*cis*-RA (9*c*RA), are mediated by a

*Received 26 March 1997*
*Revised 10 December 1997*
*Accepted 12 December 1997*

*Correspondence to:* SM Hill, Department of Anatomy, Tulane University School of Medicine, 1430 Tulane Avenue, New Orleans, LA 70112, USA

**Figure 1** Effects of melatonin and *at*RA on the proliferation of MCF-7, T47D and MDA-MB-231 cells. MCF-7 (**A**) and MDA-MB-231 (**C**) cells were seeded at a density of $2 \times 10^6$ cells $ml^{-1}$ and T47D (**B**) at $5 \times 10^5$ cells $ml^{-1}$ in Costar six-well dishes in IDMEM supplemented with 10% CS-FBS. Five hours after seeding (day 0), melatonin or *at*RA, both melatonin and *at*RA, *at*RA followed 24 h later by melatonin or melatonin followed 24 h later by *at*RA were added as 1000-fold concentrates to the appropriate wells. Ethanol vehicle was added to the control plates such that the final concentration was 0.001%. On days 1, 3 and 5, cells were harvested by brief trypsinization, and viable cells were enumerated based on trypan blue exclusion using a haemacytometer. Each point represents the mean cell count ± s.e.m. from six plates containing either the vehicle (Veh), $10^{-9}$ M melatonin (Mel), $10^{-9}$ M *at*RA (RA), melatonin plus *at*RA (M + R) or melatonin followed 24 h later by *at*RA (M24 + R) O, $^*P < 0.05$, $^{**}P < 0.01$ vs vehicle-treated controls family of nuclear retinoid receptors. This family consists of the retinoic acid receptors (RARα, RARβ, RARγ), which mediate the effects of *at*RA, and the related RXRs, which also have α, β and γ counterparts, that mediate the effects of 9*c*RA (Giguere et al, 1987; Petkovich et al, 1987; Benbrook et al, 1988; Krust et al, 1989).

Hormonal treatment of breast cancer is frequently associated with cytostatic rather than cytocidal effects, and tumours often evolve under selective pressure to become resistant to endocrine therapy in breast cancer management (Miller, 1990). The combinatory use of drugs with anti-oestrogenic effects might, thus, be an alternative to classical endocrine therapy. In this paper, we demonstrate that a sequential regimen of melatonin for 24 h followed by *at*RA results in decreased expression of ER and a significant induction of TGF-β expression, and that the combined effect of melatonin and *at*RA induces apoptosis in hormone-responsive breast tumour cells.

### MATERIALS AND METHODS

### Materials

Materials for cell culture and random priming kits were purchased from Gibco-BRL (Gaithersburg, MD, USA). 17-β Oestradiol, *at*RA and melatonin were purchased from Sigma (St Louis, MO, USA), and effector solutions were prepared in ethanol. RNAzol B reagent was purchased from Cinna-Biotex Laboratories (Houston, TX, USA), Hybond nylon transfer membrane was purchased from Amersham (Arlington Heights, IL, USA) and nitrocellulose membranes were purchased from Schleicher and Schuell (Keene, NH, USA). XOMAT-AR film was purchased from Eastman Kodak (Rochester, NY, USA). The MCF-7 and T47D cells were provided by the laboratory of the late William L McGuire (San Antonio, TX, USA), the MDA-MB-231 cells were obtained from the laboratory of CK Osborn (San Antonio) and the BT-20 cells were purchased from the American Type Tissue Culture Collection (Rockville, MD, USA).

### Cell line and culture conditions

All breast tumour cell lines, MCF-7, T47D, MDA-MB-231 and BT-20 were cultured in improved Dulbecco's modified Eagle medium (IDMEM) supplemented with 10% fetal bovine serum (FBS), 50 mM non-essential amino acids, 2 mM L-glutamine, 100 U $ml^{-1}$ penicillin, 100 μg $ml^{-1}$ streptomycin and $10^{-8}$ M porcine insulin. Experiments with melatonin and *at*RA were performed under subdued light.

### Cell proliferation studies

MCF-7 and MDA-MB-231 cells were plated at a density of $2 \times 10^6$ cells $ml^{-1}$ and BT-20 and T47D cells were plated at a density of $5 \times 10^5$ cells $ml^{-1}$ in 60-$cm^2$ wells of six-well plates in IDMEM supplemented with 10% FBS. Five hours after seeding, the cells were treated with melatonin ($10^{-9}$ M), *at*RA ($10^{-9}$ M) or a combination of the two hormones. For timed regimens, the cells were treated with either melatonin or *at*RA for 24 h before the addition of the other hormone. The cells were grown, protected from light, in a humidified atmosphere in 5% $CO_2$ at 37°C. On specific days (1, 3 or 5), triplicate wells were trypsinized, mixed with 2% trypan blue, and both total and viable cells were counted on a haemacytometer.

### DNA extraction and electrophoresis

Total cellular DNA was isolated as previously described (Nicoletti et al, 1991). Cells ($1.5 \times 10^7$) were washed and resuspended in 0.5 ml of lysis buffer (50 mM Tris, 10 mM EDTA, 0.5% Triton X-100) with 5 mg $ml^{-1}$ proteinase K and incubated at 37°C for 12 h. Lysates were then brought to a volume of 2.5 ml with lysis buffer and extracted twice with phenol–chloroform. DNA was precipitated, resuspended in 10 mM Tris (pH 8.0) with 0.1 mM EDTA and digested with 0.2 mg RNAase A for 2 h at 37°C. Approximately 20 μg of DNA from each sample was size fractionated on a 2% agarose gel, stained with ethidium bromide and photographed by UV transillumination to assess DNA oligomerization.

### Transmission electron microscopy

MCF-7 cells were cultured in IDMEM supplemented with 10% FBS and plated at a density of $1 \times 10^7$ cells in a 75-$cm^2$ flask. Cells were treated either with diluent (ethanol) or with melatonin ($10^{-9}$ M) followed 24 h later by *at*RA ($10^{-9}$ M). After 12, 24, 48 or 72 h of treatment, cells were harvested with a $Ca^{2+}$-, $Mg^{2+}$-free PBS/EDTA solution and pelleted by centrifugation (1000 ***g***) for 5 min. Cell pellets were fixed in glutaraldehyde (3%) for 1.5 h and post-fixed with osmium tetroxide (2%) for 1 h in phosphate buffer (pH 7.3). The cells were then embedded in Epox. Ultra-thin (70 nm) sections were cut with a diamond knife, stained with 5% uranyl acetate and lead citrate, and were viewed with the JEOL CX II electron microscope at 60 kV.

*© Cancer Research Campaign 1998*

**Figure 2** Electrophoretic analysis of DNA isolated from MCF-7 cells grown in IDMEM supplemented with 10% FBS was performed after treatment with the timed regimen of melatonin and *at*RA. The molecular weight marker in this figure is a combination of λ DNA digested with *Hind*III and Φ 174 digested with *Hae*III. MCF-7 cells were treated for 1, 3 or 5 days with the sequential regimen of melatonin and *at*RA (M + R), after which high molecular weight DNA was isolated as described in Materials and methods. DNA (20 µg) was run on a 2.0% agarose gel. This is a representative picture of three separate experiments

### Northern blot analysis of ER and TGF-β1 mRNA

MCF-7 cells were seeded at a density of $3.0 \times 10^6$ cells per 150-cm² flask in phenol red-free IDMEM supplemented with 5% CS-FBS. After 5 days in oestrogen-deficient medium, the cells were treated with either ethanol, melatonin ($10^{-9}$ M), *at*RA ($10^{-9}$ M) or a timed regimen of melatonin followed 24 h later by *at*RA. All treatments were continued for 24, 48 or 72 h. At the end of the treatment period, total RNA was isolated according to the method of Chomczynski and Sacchi (1987) using the RNAzol B reagent. Total RNA (50 µg) was separated electrophoretically on a 1% denaturing agarose gel containing 2.2 M formaldehyde and transferred to Hybond membranes by capillary action. The membranes were hybridized overnight with $^{32}$P-labelled ER, TGF-β, or 36B4 cDNA probes at 42°C. After high-stringency washes, filters were exposed to Kodak XOMAT-AR film with intensifying screens. Autoradiographs were scanned on the BioRad Imaging

**Figure 3** Transmission electron micrographs of MCF-7 cells following treatment with the sequential regimen of melatonin followed by *at*RA. Micrographs are representative of (**A**) control untreated cells, or cells subjected to the sequential treatment of melatonin followed by *at*RA at 12 h (**B**), 24 h (**C**), 48 h (**D**) and 72 h (**E**). The following characteristics associated with apoptosis were observed: (a) increased lysosomal bodies, (b) increased perinuclear chromatin condensation, (c) membrane and cytoplasmic blebbing, and (d) formation of membrane-bound apoptotic bodies

© Cancer Research Campaign 1998 

Densitometer GS-670 to determine the amount of ER and TGF-β1 relative to 36B4 mRNA. Results were expressed as percentages of ER and TGF-β1 mRNA levels in response to melatonin, *at*RA or the sequential regimen of melatonin and *at*RA compared with diluent controls.

### Western blot analysis of Bcl-2 and Bax expression

MCF-7 cells were treated with either diluent, melatonin, *at*RA or the sequential treatment of melatonin and *at*RA. Cultured cells were then washed twice with PBS and lysed in 300 μl $10^{-7}$ cells in 50 mM Tris (pH 8.0), 150 mM NaCl, 0.1% sodium dodecyl sulphate (SDS), 0.5% sodium deoxycholate, 0.1% Triton X, 10 μg $ml^{-1}$ phenlymethylsulphonyl fluoride, 1 μg $ml^{-1}$ aprotinin, 1 μg $ml^{-1}$ leupeptin and 0.02% sodium azide for 30 min at 4°C. Insoluble material was removed by centrifugation at 12 000 ***g*** for 15 min, and protein concentrations were determined using the BCA protein assay kit. The proteins (25 μg per lane) were size fractionated under denaturing conditions on 12.5% SDS–polyacrylamide gels and transferred to nitrocellulose membranes. Western blot analysis for Bcl-2 and Bax was conducted using rabbit polyclonal antibodies specific for the human Bcl-2 and Bax proteins as previously described (Krajewski et al, 1995) and an actin rabbit polyclonal antibody (Sigma) as a loading control. The proteins were visualized after incubation with horseradish peroxidase-conjugated secondary antibody (Sigma) and chemiluminescent substrate (Amersham), and exposure to Kodak XOMAT-AR film. Autoradiographs were scanned densitometrically to determine the amount of Bcl-2 and Bax proteins relative to the actin protein. Results were normalized to actin protein levels and are expressed as per cent of each day's individual control.

### RESULTS

### A sequential regimen of melatonin followed by *at*RA is cytocidal in ER-positive MCF-7 and T47D but not in ER-negative MDA-MB-231 or BT-20 human breast cancer cells

In light of recent reports that melatonin may be a ligand for the RORα receptors, that both melatonin and *at*RA can suppress the growth of ER-positive breast cancer cells (Lacroix and Lippman, 1980; Hill and Blask, 1988) and that the RORα and RARα receptors may crosstalk at the level of hormone response element, we initiated a series of studies to examine the possible additive or synergistic effects of melatonin and retinoic acid. As shown in Figure 1A, ER-positive MCF-7 breast tumour cells showed significant growth suppression to 64% and 62% of control after 5 days of either melatonin ($10^{-9}$ M) or *at*RA ($10^{-9}$ M) treatment respectively. Surprisingly, the simultaneous treatment of cells with melatonin and *at*RA had no inhibitory effect on cell proliferation, and these cell numbers were equivalent to control values. However, a sequential regimen of melatonin ($10^{-9}$ M) followed 24 h later by *at*RA ($10^{-9}$ M) resulted in a cytocidal effect, decreasing cell numbers to below the initial plating density after 5 days of treatment. Sequential treatment with retinoic acid followed 24 h later by melatonin had a cytostatic effect in which cell proliferation was inhibited to 48% of control, but not a cytocidal effect. Similar results were seen with the ER-positive T47D cell line (Figure 1B) in which melatonin and *at*RA, when used alone, inhibited cell proliferation to 60% and 61% of control respectively; however, the simultaneous

**Figure 4** Effects of treatment with melatonin or *at*RA alone vs the sequential regimen of melatonin and *at*RA on steady-state ER and TGF-β1 mRNA levels in MCF-7 cells cultured in medium supplemented with 5% CS-FBS. MCF-7 cells were incubated with ethanol diluent (control), $10^{-9}$ M melatonin (Mel), $10^{-9}$ M *at*RA (*at*RA) or a regimen of melatonin followed 24 h later by *at*RA (M + R). For each time point, 50 μg of total RNA was fractionated on denaturing 1% agarose gels and blotted as described in Materials and methods. Northern blots were probed with $^{32}$P-labeled human ER and human TGF-β1 cDNAs. The 36B4 cDNA was used to monitor RNA loading. A representative autoradiograph is shown in (**A**). Autoradiographs from Northern blot analyses were quantified by scanning densitometry and normalized to 36B4 mRNA. Results are presented graphically in (**B**) as per cent of control (*n* = 3 independent experiments). **P* < 0.001 vs controls, ***P* < 0.005 vs melatonin *or at*RA alone

© Cancer Research Campaign 1998

administration of melatonin and *at*RA also inhibited cell growth (54% vs control). T47D cells, like MCF-7 cells, showed a cytocidal response to the sequential regimen of melatonin and *at*RA. Sequential treatment with retinoic acid followed 24 h later by melatonin caused a cytostatic effect in which cell proliferation was inhibited to 39% of control, but a cytocidal effect was not observed. The specificity of the cytocidal effects is demonstrated by the fact that the sequential melatonin and *at*RA regimen had no effect on ER-negative MDA-MB-231 breast cancer cells (Figure 1C) nor on ER-negative BT-20 cells (data not shown).

### Electrophoretic analysis of DNA isolated from MCF-7 cells treated at various times with the sequential regimen of melatonin and *at*RA

The pattern of DNA oligomerization in MCF-7 tumour cells was determined at various times following the initiation of the sequential regimen of melatonin followed by *at*RA (Figure 2). Following treatment, the development, over time, of a ladder of nucleosomal oligomers was evident in MCF-7 cells. This laddering is characteristic of many cell types undergoing apoptosis. It should also be noted that there was no evidence of complete DNA degradation, which would be expected if the cells were undergoing cellular necrosis in response to treatment with melatonin followed by *at*RA.

### Morphological changes in MCF-7 cells treated with the sequential regimen of melatonin and *at*RA

Apoptosis is delineated from cellular necrosis by a unique series of ultrastructural changes, including chromosomal and cytoplasmic condensation, nuclear fragmentation, membrane blebbing, increased number of lysosomal bodies and the formation of membrane-bound apoptotic bodies. Following treatment with the sequential regimen of melatonin and *at*RA, MCF-7 cells were examined for ultrastructural changes indicative of necrosis or apoptosis. No distinctive morphological changes were noted in cells treated with the sequential regimen of melatonin and *at*RA until after 24 h of treatment, at which time an increase in lysosomal bodies, and perinuclear chromatin condensation was observed as compared with controls (Figure 3A–C). By 48 h, there was a further increase in the presence of lysosomal bodies and perinuclear chromatin condensation, and the cells had begun to demonstrate membrane blebbing (Figure 3D). After 72 h of treatment, membrane and cytoplasmic blebbing had increased and membrane-bound apoptotic bodies were evident. Based on the morphological criteria, it is evident that the treatment of MCF-7 cells with melatonin followed by *at*RA induced apoptosis rather than necrosis.

### Effects of melatonin and *at*RA on the expression of the steady-state levels of ER and TGF-β mRNAs

The expression of the steady-state levels of mRNAs encoding the ER and TGF-β1 was examined by Northern blot analysis in MCF-7 cells following 48 h of treatment with melatonin or *at*RA alone or with the sequential regimen of melatonin and *at*RA. For these studies, MCF-7 cells were grown for 5 days in oestrogen-deficient medium and treated with either melatonin or *at*RA alone ($10^{-9}$ M) or pretreated with melatonin for 24 h before the addition of *at*RA. Figure 4A shows that both *at*RA and melatonin alone significantly decreased the steady-state level of ER mRNA by 62% and 79%

**Figure 5** The temporal effects of the sequential regimen of melatonin and *at*RA on steady-state TGF-β1 mRNA expression in MCF-7 cells. MCF-7 cells were cultured in medium supplemented with 5% CS-FBS. MCF-7 cells were incubated with diluent (ethanol), or a regimen of melatonin followed 24 h later by *at*RA for 1, 2 or 3 days. For each time point, 50 μg of total RNA was fractionated on denaturing 1% agarose gels and blotted as described in Materials and methods. Northern blots were probed with $^{32}P$-labeled human TGF-β1 cDNAs. The 36B4 cDNA was used to monitor RNA loading. A representative autoradiograph is shown in (**A**). Autoradiographs from Northern blot analyses were quantified by scanning densitometry and normalized to 36B4 mRNA ($n$ = 3 independent experiments). Results are presented graphically as per cent of control (**B**). $^{*}P < 0.001$ vs controls, $^{**}P < 0.05$ vs day 1 or 2 of treatment

© Cancer Research Campaign 1998

respectively ($P < 0.01$ vs control). However, the sequential regimen of melatonin and *at*RA reduced ER mRNA expression to almost undetectable levels ($P < 0.001$ vs melatonin or *at*RA alone). In addition, both *at*RA and melatonin alone enhanced the steady-state level of TGF-β1 mRNA by 40% and 53% respectively, whereas the sequential regimen produced a super-induction of TGF-β1 mRNA levels (91% increase over control, and 65% and 52% increase over *at*RA or melatonin treatment respectively).

Figure 5A shows a representative Northern blot analysis of the time course of TGF-β1 mRNA expression in response to the sequential treatment of MCF-7 cells with melatonin followed by *at*RA. By days 1 and 2 of treatment, TGF-β1 mRNA levels were increased 30% and 70%, respectively, over controls (Figure 5B). By day 3, TGF-β1 mRNA was markedly elevated to approximately 200% over diluent controls. These results suggest that the sequential treatment of MCF-7 cells with melatonin followed by *at*RA results in a synergistic induction of TGF-β1 mRNA expression.

### Temporal expression of Bcl-2 and Bax in MCF-7 cells in response to the timed treatment of melatonin and *at*RA

The relative levels of the Bcl-2 and Bax proteins were examined by Western blot analysis. Figure 6A and B shows representative Western blots of Bcl-2 and Bax expression 5 days after pretreatment with melatonin followed 24 h later by *at*RA. Densitometric analysis of these and other Western blots is shown in Figure 7. The levels of Bcl-2 exhibited the greatest divergence from control levels on days 3 and 4, at which time Bcl-2 expression was reduced by 64% and 66% respectively as compared with control. Conversely, Bax expression appeared to decrease as compared with controls on day 4; however, this decrease was not statistically significant. On day 5, however, Bax expression was significantly increased over control by 58%. These results suggest that the sequential treatment regimen of melatonin followed by *at*RA suppresses the expression of the 'death suppresser', Bcl-2, on days 2, 3, 4 and 5 and enhances the expression of the 'death inducer', Bax, only on day 5.

### DISCUSSION

Previous work by our laboratory (Hill and Blask, 1988), as well as that of others (Cos and Sánchez-Barceló, 1994), has clearly demonstrated the growth-inhibitory effect of melatonin on human breast tumour cells. In addition, numerous laboratories have reported that both *at*RA and 9*c*RA are effective inhibitors of breast tumour cell proliferation (Lotan, 1979; Lacroix and Lippman,

**Figure 6** The temporal effects of the sequential regimen of melatonin and *at*RA on Bcl-2 and Bax protein expression in MCF-7 cells. MCF-7 cells were incubated with diluent (control) or a regimen of melatonin followed 24 h later by *at*RA (M + *at*RA) for 1, 2, 3, 4 or 5 days. For each time point, 25 μg of total cellular protein per lane was fractionated on 12.5% polyacrylamide gels and transferred to nitrocellulose membranes as described in Materials and methods. Western blots were probed with polyclonal antibodies specific for Bcl-2 and Bax, and proteins were visualized after incubation with a horseradish peroxidase-conjugated secondary antibody and chemiluminescent substrate. Actin protein levels were used to monitor protein loading. Representative Western blots for Bcl-2 (**A**) and Bax (**B**) during 5 days of treatment with melatonin followed 24 h later by *at*RA are shown

 © Cancer Research Campaign 1998

**Figure 7** Time-course changes in Bcl-2 and Bax protein levels after sequential treatment of MCF-7 cells with melatonin followed by *at*RA. Autoradiographs from Western blot analyses of the time course of Bax (solid bars) and Bcl-2 (open bars) proteins in response to the sequential treatment of melatonin and *at*RA were quantified by scanning densitometry and normalized to actin protein levels. Results are presented graphically as per cent of control (*n* = 3 independent experiments). *$P < 0.05$ vs controls

1980; Fontana et al, 1990, 1991; van der Burg et al, 1993; Rubin et al, 1994). Although each hormone has been shown to slow tumour proliferation, neither hormone alone has been shown to produce cytocidal effects in breast cancer cells at physiological concentrations. However, the results presented here show that, when used in a sequential manner (melatonin followed 24 h later by *at*RA), these hormones are able to act in an additive or synergistic manner to induce a cytocidal response and apoptosis in hormone-responsive breast tumour cells. For example, the formation of nucleosomal DNA oligomers, a phenomenon seen in many cells undergoing programmed cell death, was evident after treatment of MCF-7 cells with the timed regimen of melatonin and *at*RA. Furthermore, based on the morphological criteria of chromosomal condensation, nuclear fragmentation, membrane blebbing, increased number of lysosomal bodies and the formation of membrane-bound apoptotic bodies, it was clear that MCF-7 cells were undergoing apoptosis rather than necrosis in response to this treatment paradigm. The cytocidal effects produced by the sequential regimen of melatonin and *at*RA do not appear to result from non-specific cytotoxic effects, but, rather, are probably cell and treatment specific. This is evident by the lack of response of ER-negative (hormone-insensitive) MDA-MB-231 and BT-20 cells to the sequential regimen, and by the failure of the simultaneous administration of melatonin and *at*RA to induce apoptosis. However, as we did not specifically quantify the number of cells undergoing apoptosis vs those undergoing a more general cytocidal response, we cannot definitively say that apoptosis was the primary contributor to this cytocidal effect.

An interesting observation was that simultaneous treatment of MCF-7 cells with melatonin and *at*RA did not suppress cell proliferation, but rather cell numbers were equivalent to control values. This may be due to a time-dependent response in which melatonin may potentiate the effects of retinoic acid, possibly through either up-regulation or phosphorylation of the retinoic acid receptor or through modulation of the ER. Studies are currently under way in our laboratory to investigate these possibilities. The observation that simultaneous treatment of MCF-7 cells with melatonin and *at*RA failed to suppress cell proliferation is in contrast to the effect seen in T47D cells, in which the simultaneous use of melatonin and *at*RA did inhibit cell proliferation, but not to any greater degree than either hormone alone. The results also show that, whereas a sequential treatment regimen of retinoic acid followed 24 h later by melatonin results in a cytostatic effect on cell proliferation, a cytocidal response is seen only with the sequential treatment of melatonin followed 24 h later by retinoic acid. Studies are currently being conducted to better characterize this sequence-dependent effect, as well as the antagonistic response that occurs when melatonin and retinoic acid are administered simultaneously. These observations suggest that there is some level of crosstalk between the melatonin and RA signalling pathways and that the signalling pathways between the MCF-7 and T47D cell lines may be somewhat different. Most evidence suggests that the effects of melatonin are mediated via membrane-associated receptors, two of which ($Mel_{1a}$ and $Mel_{1b}$) have recently been cloned (Ebisawa et al, 1994; Reppert et al, 1995). Although controversial, some reports have also suggested that melatonin is able to bind and activate nuclear RORα receptors (Becker-André et al, 1994; Steinhilber et al, 1995). Transcripts for both of these receptors ($Mel_{1a}$ and RORα) are expressed in MCF-7 cells (manuscript in preparation). This is an interesting observation as we have found that transcripts for the RORα2, 3 and 4 mRNAs, but not RORα1, are expressed in MCF-7 but not in MDA-MB-231 cells (Ram and Hill, 1995). If melatonin's effects are mediated via the membrane receptors, it is possible that cross-talk could occur between the melatonin and RA signalling pathways via phosphorylation of RAR or RXR receptors. However, crosstalk between the RORα and RAR/RXR receptors has already been demonstrated at the level of the hormone response element (Tini et al, 1995).

Expression of the 'death suppresser', Bcl-2 has previously been shown to be up-regulated by oestrogens in MCF-7 cells (Teixeira et al, 1995), a process clearly mediated via the ER. The significant diminution of ER mRNA levels in response to the sequential regimen of melatonin and *at*RA raises the possibility that the down-regulation of Bcl-2 expression by this treatment is mediated indirectly via the reduction in ER expression. However, the lack of a 1:1 correlation between the per cent reduction in the levels of ER and Bcl-2 suggests that this treatment regimen may involve additional pathways that modulate Bcl-2 expression. Another potential contributor to these apoptotic effects could be the up-regulation of Bax protein seen on day 5 of the sequential treatment with melatonin and *at*RA. However, as Bax expression on day 4 appeared to decrease (not significantly) compared with controls, and as the increase in Bax expression observed on day 5 appeared much later than the onset of apoptosis in these cells, it is probable that this is a secondary effect rather than a primary contributor to the onset of apoptosis and must be interpreted with caution. It may be that the overall ratio of the 'death suppresser' (Bcl-2) to the 'death inducer' (Bax) is more important in mediating the apoptotic effects than is either one alone.

It is also possible that Bcl-2- and Bax-associated pathways play a secondary role, and that the overexpression of TGF-β1 induced by this sequential melatonin and *at*RA treatment is the critical event leading to apoptosis. TGF-β1 has been shown to be a potent growth inhibitor of breast epithelium and breast tumour cells, particularly MCF-7 cells (Arteaga et al, 1990). It is also known

© Cancer Research Campaign 1998

that oestrogen down-regulates TGF-β1 expression and secretion in MCF-7 cells, and that the antioestrogen tamoxifen can block oestrogen's suppressive effect and promote enhanced TGF-β1 synthesis and secretion (Knabbe et al, 1987). We have previously demonstrated that melatonin can up-regulate TGF-β1 mRNA expression in a time course independent of the effects of oestrogen (Molis et al, 1995). Thus, for the sequential treatment in which super-induction of TGF-β1 expression occurs, two mechanisms are possible. First, the effects of this regimen may be mediated solely through the down-regulation of the oestrogen-response pathway and the secondary up-regulation of TGF-β1 levels. Alternatively, the effects on TGF-β1 may be mediated by multiple pathways, including the oestrogen-response pathway. Studies are currently under way to determine if this sequential regimen of melatonin and *at*RA can induce apoptosis in the face of experimentally achieved reductions in TGF-β1 levels.

Both melatonin and RA can inhibit the proliferation of various malignant cell types, including breast cancer. However, only retinoids have been shown to induce cell death and often when employed at supraphysiological concentrations (Lacroix and Lippman, 1980). Thus, at least for RA, the major drawback to its use as a therapeutic agent is the toxicity induced at pharmacological doses. For this reason, the development of combinatorial therapies which would reduce the concentrations needed for clinical efficacy yet still enhance anti-tumorigenic activity would be of great benefit. Our data indicate that the antiproliferative effects of melatonin and RA on human breast cancer cells may be additive or synergistic when administered in the appropriate order, time and dose. In fact, when used in a given paradigm, they may be able to induce specifically apoptosis of breast tumour cells and the regression of breast tumours. This treatment regimen has been tested only in cultured breast cancer cells; therefore, the effects on primary human breast tumours or cell lines grown as xenografts may be very different from the effects observed in our long established MCF-7 breast cancer cell line. Our laboratory currently has studies in progress to investigate further the potential effects of our sequential treatment regimen on *N*-nitroso-*N*methylurea (NMU)-induced mammary tumours in the rat animal model. In order to define the optimal treatment parameters to induce tumour regression in vivo, a more detailed understanding of the biochemical pathway(s) involved in programmed cell death of breast tumour cells is needed.

### REFERENCES

Arteaga CL, Coffey RJ, Dugger TC, McCutchen CM, Moses HL and Lyons RM (1990) Growth stimulation of human breast cancer cells with anti-transforming growth factor β antibodies: evidence for negative autocrine growth regulation by transforming growth factor β. *Cell Growth Differ* **1**: 367–374

Bacus SS, Kiguchi K, Chin D, King CR and Huberman E (1990) Differentiation of cultured human breast cancer cells (AU-565 and MCF-7) associated with loss of cell surface Her-2/neu antigen. *Mol Carcinog* **3**: 350–362

Becker-André M, Weisenberg I, Schaeren-Wiemers N, André E, Missbach M, Saurat J-H and Carlberg C (1994) Pineal gland hormone melatonin binds and activates an orphan of the nuclear receptor superfamily. *J Biol Chem* **269**: 28531–28534

Benbrook D, Lernhardt E and Pfahl M (1988) A new retinoic acid receptor identified from a hepatocellular carcinoma. *Nature (Lond)* **333**: 669–672

Blask DE, Hill SM, Orstead KM and Massa JS (1986) Inhibitory effects of the pineal hormone melatonin and underfeeding during the promotional phase of 7,12-dimethylbenzantracene (DMBA)-induced mammary tumorigenesis. *J Neural Transm* **67**: 125–138

Blask DE, Pelletier DB, Hill SM, Lemus-Wilson A, Grosso DS, Wilson ST and Wise ME (1991) Pineal melatonin inhibition of tumor promotion in N-nitroso-N-methylurea model of mammary carcinogenesis: potential involvement of antiestrogenic mechanisms in vivo. *J Cancer Res Clin Oncol* **117**: 526–532

Chomczynski P and Sacchi N (1987) Single step method of RNA isolation by acid guanidinium thiocyanate-phenol-chloroform extraction. *Anal Biochem* **162**: 156–159

Clarke CL, Roman SD, Graham J, Koga M and Sutherland RL (1990) Progesterone receptor regulation by retinoic acid in the human breast cancer cell line T-47D. *J Biol Chem* **265**: 12694–12700

Cos S and Sánchez-Barceló EJ (1994) Differences between pulsatile or continuous exposure to melatonin on MCF-7 human breast cancer cell proliferation. *Cancer Lett* **85**: 105–109

Ebisawa T, Karne S, Lerner MR and Reppert SM (1994) Expression cloning of a high-affinity melatonin receptor from Xenopus dermal melanophores. *Proc Natl Acad Sci USA* **91**: 6133–6137

Fontana JA, Mezu AB, Cooper BN and Miranda D (1990) Retinoid modulation of estradiol-stimulated growth and of protein synthesis and secretion in human breast carcinoma cells. *Cancer Res* **50**: 1997–2002

Fontana JA, Burrows-Mezu A, Clemmons DR and LeRoith D (1991) Retinoid modulation of insulin-like growth factor binding proteins and inhibition of breast carcinoma proliferation. *Endocrinology* **128**: 1115–1122

Giguere V, Ong ES, Segui P and Evans RM (1987) Identification of a receptor for the morphogen retinoic acid. *Nature (Lond)* **330**: 624–629

Hill SM and Blask DE (1988) Effects of the pineal hormone melatonin on the proliferation and morphological characteristics of human breast cancer cells (MCF-7) in culture. *Cancer Res* **48**: 6121–6129

Knabbe C, Lippman ME, Wakefield LM, Flanders KC, Kasid A, Derynck R and Dickson RB (1987) Evidence that transforming growth factor beta is a hormonally regulated negative growth factor in human breast cancer. *Cell* **48**: 417–423

Krajewski S, Blomqvist C, Franssila K, Krajewsi M, Wasenius VM, Niskanen E, Nordling S and Reed JC (1995) Reduced expression of proapoptotic gene Bax is associated with poor response rates to combination chemotherapy and shorter survival in women with metastatic breast adenocarcinoma. *Cancer Res* **55**: 4471–4478

Krust A, Kastner P, Petkovich M, Zelent A and Chambon P (1989) A third human retinoic acid receptor, hRARγ. *Proc Natl Acad Sci USA* **86**: 5310–5314

Lacroix A and Lippman ME (1980) Binding of retinoids to human breast cancer cell lines and their effects on cell growth. *J Clin Invest* **65**: 586–591

Lotan R (1979) Different susceptibilities of human melanoma and breast carcinoma cell lines to retinoic acid-induced growth inhibition. *Cancer Res* **39**: 1014–1019

Miller WR (1990) Endocrine treatment for breast cancers: biological rationale and current progress. *J Steroid Biochem Mol Biol* **37**: 467–480

Molis TM, Spriggs LL and Hill SM (1994) Modulation of estrogen receptor mRNA expression by melatonin in MCF-7 human breast cancer cells. *Mol Endocrinol* **8**: 1683–1690

Molis TM, Spriggs LL, Jupiter Y and Hill SM (1995) Melatonin modulation of estrogen-regulated proteins, growth factors, and proto-oncogenes in human breast cancer. *J Pineal Res* **18**: 93–103

Nicoletti I, Migliorati G, Paglancci M, Grignani F and Riccardi C (1991) A rapid and simple method for measuring thymocyte apoptosis by propidium iodide staining and flow cytometry. *J Immunol Methods* **139**: 271–279

Petkovich M, Brand NJ, Krust A and Chambon P (1987) A human retinoic acid receptor which belongs to the family of nuclear orphan receptors. *Nature (Lond)* **330**: 444–450

Ram PT and Hill SM (1995) Melatonin's inhibition of breast cancer cell proliferation is mediated through the RORα receptor pathway. 5th Int. Congress on Hormones and Cancer, abstract 73, p. 107, Quebec, Canada

Reppert SM, Godson C, Mahle CD, Weaver DR, Slaugenhaupt SA and Gusella JF (1995) Molecular characterization of a second melatonin receptor expressed in human retina and brain: the $Mel_{1b}$ melatonin receptor. *Proc Natl Acad Sci USA* **92**: 8734–8738

Rubin M, Fenig E, Rosenauer A, Menendez-Botet C, Achkar C, Bentel JM, Yahalom J, Mendelsohn M and Miller WH (1994) 9-cis Retinoic acid inhibits growth of breast cancer cells and down-regulates estrogen receptor RNA and protein. *Cancer Res* **54**: 6549–6556

Steinhilber D, Brungs M, Werz O, Wiesenberg I, Danielsson C, Kahlen J-P, Nayeri S, Schräder M and Carlberg C (1995) The nuclear receptor for melatonin represses 5-lipoxygenase gene expression in human B lymphocytes. *J Biol Chem* **270**: 7037–7040

Teixeira C, Reed JC and Pratt MAC (1995) Estrogen promotes chemotherapeutic drug resistance by a mechanism involving Bcl-2 proto-oncogene expression in human breast cancer cells. *Cancer Res* **55**: 3902–3907

© Cancer Research Campaign 1998

Tini M, Fraser RA and Giguère V (1995) Functional interactions between retinoic acid receptors-related orphan nuclear receptor (RORα) and the retinoic acid receptors in the regulation of the γF-crystalline promoter. *J Biol Chem* **270**: 20156–20161

van der Burg B, van der Leede B-JM, Kwkkenbos-Isbrucker L, Salverda S, de Laat SW and van der Saag PT (1993) Retinoic acid resistance of estradiol-independent breast cancer cells coincides with diminished retinoic acid receptor function. *Mol Cell Endocrinol* **91**: 149–147

© Cancer Research Campaign 1998